# Acute Invasive Fungal Rhinosinusitis: Insights on Mortality and Surgical Outcomes From a Cohort Study

**DOI:** 10.7759/cureus.91843

**Published:** 2025-09-08

**Authors:** António Andrade, Patrícia S Sousa, João C Almeida, Ricardo Vaz, Gil Coutinho

**Affiliations:** 1 Department of Otorhinolaryngology, Unidade Local de Saúde de São João, Porto, PRT; 2 Unit of Otorhinolaryngology, Department of Surgery and Physiology, Faculty of Medicine, University of Porto, Porto, PRT; 3 Department of Otorhinolaryngology, Unidade Local de Saúde de Trás-os-Montes e Alto Douro, Vila Real, PRT; 4 Department of Pathology, Unidade Local de Saúde de São João, Porto, PRT; 5 Unit of Anatomy, Department of Biomedicine, Faculty of Medicine, University of Porto, Porto, PRT; 6 CINTESIS@RISE (Center for Health Technology and Services Research, RISE Health), Faculty of Medicine, University of Porto, Porto, PRT; 7 NeuroGen Research Group, Center for Health Technology and Services Research (CINTESIS), Porto, PRT

**Keywords:** hyperbaric oxygen therapy, immunocompromised host, invasive fungal infections, orbital exenteration, rhinosinusitis

## Abstract

Introduction

Acute invasive fungal rhinosinusitis (AIFRS) is a rapidly progressive infection that primarily affects immunocompromised patients. This study aims to characterize the clinical and microbiological findings and assess treatment outcomes in cases with AIFRS.

Material and methods

A retrospective observational study was conducted between January 2013 and July 2024. Patients were included based on histopathological confirmation of fungal infection and clinical evidence of AIFRS. Data on demographics, comorbidities, symptoms, imaging, microbiology, treatment, and outcomes were collected.

Results

Thirteen patients were included, with a mean age of 49.7 (±15.9) years and a predominance of female sex (10/13). Diabetes mellitus and hematologic malignancies were the most common comorbidities (six and five cases, respectively). The most frequent pathogens were *Aspergillus *spp. and *Mucorales *spp. (six cases each). The sphenoid and ethmoid sinuses were the most commonly affected (11 cases each). Orbital invasion and cavernous sinus involvement were observed in 10 and five cases, respectively. Four patients underwent orbital exenteration and survived. Three patients, with distinct underlying etiologies and fungal pathogens, were treated concomitantly with hyperbaric oxygen therapy, with no reported fatalities. The overall mortality rate was 53.8%, with 38.5% directly attributed to AIFRS.

Conclusion

AIFRS, predominantly caused by *Aspergillus *spp. or *Mucorales *spp*, *is associated with high mortality, particularly in immunocompromised patients. Orbital involvement was frequent, and exenteration was performed in selected cases. Hyperbaric oxygen therapy was used as an adjunctive treatment in three patients, all of whom survived. These findings highlight the need for further studies to clarify therapeutic decision-making in AIFRS.

## Introduction

Fungal nasosinusal infections can be characterized by their clinical course, histopathological features, and immune response to the pathogen [1]. Acute invasive fungal rhinosinusitis (AIFRS) is notable for its aggressive, rapid progression, typically within one month, and demonstrates histopathological patterns of severity [1,2]. This aggressive behavior involves mucosal invasion, bone erosion, and vascular involvement, culminating in hemorrhage, thrombosis, infarction, and tissue necrosis.

The most common pathogens are *Aspergillus* spp. and *Mucorales* spp., with histopathological findings showing hyphal invasion in infected tissue [2]. These infections typically occur in immunocompromised patients, where the immune system is unable to effectively control the pathogen. Immunosuppression may result from hematologic malignancies with neutropenia, poorly controlled diabetes mellitus (DM), acquired immunodeficiency syndrome (AIDS), or immunosuppressive therapies such as chemotherapy, corticosteroids, or other immunosuppressants [1,3]. Often, these patients present with multiple risk factors, reflecting the complexity of modern medicine.

Although rare, AIFRS carries significant clinical relevance due to its high morbidity and mortality, with fatality rates ranging between 50% and 80% [1,3]. This underscores the need for early diagnosis, a high index of suspicion, and prompt treatment to improve survival outcomes. Moreover, given the increasing incidence of immunosuppression through pharmacology and the extended survival of immunocompromised patients [4], it is crucial to identify cases of AIFRS to optimize management strategies. Therefore, the aim of this study was to comprehensively characterize the clinical and microbiological features of patients with histologically and clinically confirmed AIFRS, to describe imaging findings, to assess surgical and medical management strategies, including adjunctive therapies, and to analyze patient outcomes.

This article was previously posted to Research Square preprint platform on March 26, 2025.

## Materials and methods

A retrospective observational study was conducted in the Department of Otorhinolaryngology, Unidade Local de Saúde de São João, Porto, Portugal, between January 2013 and July 2024. Cases were identified through histopathological confirmation of fungal infection in patients whose clinical history supported the diagnosis of AIFRS [2]. The study was approved by the Ethics Committee of Unidade Local de Saúde de São João (approval number: CE-347-2024). All procedures followed the regulations established by the Ethics and Clinical Research Committee and the Helsinki Declaration of the World Medical Association

Participants

Pediatric cases were excluded (age under 18 years). Only adult patients with confirmed histopathological evidence of invasive fungal infection and compatible clinical presentation between January 2013 and July 2024 were included. All patients underwent sinonasal surgery. Postoperatively, daily evaluations were performed via endoscopic examination, with debridement under local anesthesia as required. Antifungal dosages were individualized based on the isolated microbiological agents and patient comorbidities.

Data collection

All data were retrieved from the electronic medical records. Data collected included demographics (age, gender), comorbidities, presenting symptoms, and microbiological culture results. Computed tomography (CT) and magnetic resonance imaging (MRI) findings were used to assess involvement of the maxillary, ethmoidal, frontal, and sphenoidal sinuses, as well as the intracranial, cavernous sinus, orbital, skin, and palate regions. Additionally, medical treatment, surgical approaches (endoscopic sinus surgery, cranioendoscopic approach, and orbital exenteration), and mortality were recorded. Mortality directly related to AIFRS was defined as death occurring as a direct consequence of fungal infection or its complications, as determined by the treating multidisciplinary team. Diagnostic and treatment protocols remained consistent throughout the study period. Initial antifungal therapy involved broad-spectrum agents and was subsequently tailored according to the isolated pathogen. Surgical decisions and the use of hyperbaric oxygen therapy (HOT) were made on a case-by-case basis in multidisciplinary meetings, considering clinical status, infection extent, treatment response, and patient preference. Surgical indication was based on functional preservation whenever possible, avoiding unnecessary aggressive procedures. 

Statistical analyses

Statistical analysis was performed using IBM SPSS Statistics for Windows, version 27 (Released 2019; IBM Corp., Armonk, New York, United States). Categorical variables were examined through absolute and relative frequencies. The Kolmogorov-Smirnov test was employed to determine the normality of continuous variables. Continuous variables with normal distributions were analysed using mean and standard deviation. 

## Results

Thirteen cases were included in the study. Table 1 presents an overview of patient characteristics. The mean age was 49.7 years (±15.9), normally distributed, with a predominance of female patients (n=10; 76.9%). Regarding immunosuppression-related comorbidities, there was a significant overlap of conditions, with DM (n=6) and hematologic malignancies (n=5) being the most prevalent. These malignancies included acute myeloid leukemia, dendritic cell blast neoplasm, Burkitt’s leukemia, small lymphocytic lymphoma, and idiopathic CD4 lymphopenia. Pharmacological immunosuppression was identified in four patients due to rheumatologic disease. One case involved a patient with AIDS, and another involved a breast cancer diagnosis without the initiation of chemotherapy.

**Table 1:** Characteristics of the participants (N=13)

Characteristics	Frequency (Percentage)
Female sex	10 (76.9%)
Comorbidities	
Diabetes mellitus	6 (46.2)
Hematological disorders	5 (38.5)
Imunossupresive therapy	4 (30.8)
AIDS	1 (7.7)
Breast cancer	1 (7.7)
Extension	
Ethmoid sinus	11 (84.6)
Sphenoid sinus	11 (84.6)
Maxilar sinus	9 (69.2)
Frontal sinus	2 (15.4)
Orbital cavity	10 (76.9)
Intracranial	8 (61.5)
Cavernous sinus	5 (38.5)
Hard palate	1 (7.7)
Skin	1 (7.7)
Fungal Infection	
*Mucor *spp	6 (46.2)
*Aspergillus *spp	6 (46.2)
*Scedosporium *spp	1 (7.7)

The most common presenting symptoms were headache and facial pain, observed in 10 (76.9%) and eight (61.5%) patients, respectively, followed by ophthalmologic signs such as visual impairment (n=7; 53.8%) and ophthalmoplegia (n=6; 46.2%). Notably, none of the patients presented with fever. Involvement of the paranasal sinuses was also assessed, with the sphenoid and ethmoid sinuses being the most frequently affected (n=11 each; 84.6%), followed by the maxillary sinus in nine (69.2%) patients and the frontal sinus in two (15.4%) patients. Orbital and intracranial extension was frequent, occurring in 10 (76.9%) and eight (61.5%) patients, respectively. Five (38.5%) patients showed cavernous sinus involvement, while one (7.7%) patient extended to the palate and another to the skin.

Microbiological cultures identified *Aspergillus* spp. in six (46.2%) patients, *Mucor* spp. in six (46.2%) patients, and *Scedosporium boydii *in* *one (7.7%) patient. All patients underwent sinonasal surgery. Four (30.8%) patients required orbital exenteration, and two (15.4%) had cranial involvement requiring surgical intervention. Antifungal therapies used during hospitalization are detailed in Table 2. Three (23.1%) patients underwent hyperbaric oxygen therapy (HOT), with no reported fatalities in these cases.

**Table 2:** Patient management and mortality rates (N=13) AIFRS: acute invasive fungal rhinosinusitis

Parameters	Frequency (Percentage)
Multidisciplinary surgery	
Orbital exenteration	4 (30.8)
Neurosurgical approach	2 (15.4)
Hyperbaric oxygen therapy	3 (23.1)
Antifungal therapy	
Amphotericin	13 (100)
Isavuconazole	6 (46.2)
Posaconazole	4 (30.8)
Voriconazole	4 (30.8)
Caspofungin	2 (15.4)
Fluconazole	2 (15.4)
Micafungin	1 (7.7)
Mortality	
Overall	7 (53.8)
Directly related to AIFRS	5 (38.5)

The overall mortality rate was 53.8%, with seven fatal cases. Five deaths were directly related to AIFRS (38.5%). One death resulted from progression of the underlying hematologic malignancy, and another was due to acute myocardial infarction in a patient with a history of DM. A detailed list of patient data is presented in Table 3.

**Table 3:** Detailed patient data AIFRS: acute invasive fungal rhinosinusitis; BC: breast cancer; CS: cavernous sinus; DM: diabetes mellitus; F: female; HO: hematological disorders; HOT: hyperbaric oxygen therapy; IC: intracranial extension; IT: Immunosuppressive therapy; M: male; MI: myocardial infarction; NS: neurosurgery

Sex	Age (years)	Diagnosis year	Comorbidities	Fungus	IC	CS	Orbital	Orbital exenteration	NS	HOT	Death
M	54	2013	DM, IT, HO	Mucor	✓		✓				AIFRS
F	46	2013	AIDS	Mucor							
F	52	2013	HO	Mucor	✓		✓				AIFRS
F	29	2013	HO	Aspergillus							AIFRS
F	39	2013	DM	Mucor	✓	✓	✓	✓			MI
M	73	2017	HO	Aspergillus	✓	✓	✓			✓	
F	33	2018	HO	Aspergillus			✓				HO
F	19	2018	DM	Mucor		✓	✓	✓		✓	
F	54	2020	BC	Scedosporium	✓	✓	✓				AIFRS
M	68	2022	DM, IT	Aspergillus	✓		✓		✓		AIFRS
F	61	2022	DM, IT	Mucor	✓		✓	✓	✓	✓	
F	55	2023	IT	Aspergillus							
F	63	2024	DM	Aspergillus	✓	✓	✓	✓			

## Discussion

Our study highlights the complexity of AIFRS management and the necessity for a high index of suspicion, particularly in immunocompromised patients who may present without fever, underscoring the importance of recognizing localized symptoms, such as facial pain and ophthalmologic signs, for early diagnosis and intervention. The most frequent symptoms in our sample were headache and facial pain, consistent with the literature [5]. Fever was absent in all patients, despite being commonly reported in the literature as a presenting symptom [1,4]. In immunocompromised patients, symptoms may present later in the disease course, making clinical signs more significant. Immunosuppression often dampens the inflammatory response, and in cases of DM, neuropathy may delay the onset of pain, leading to late presentation [6]. Ophthalmologic signs were among the most significant clinical findings. The presence of ophthalmoplegia, visual impairment, and ocular pain is a strong indicator of orbital involvement in fungal infections [6]. The spread of infection to the orbit can occur via two main routes [7,8]: direct invasion of adjacent tissues, such as the lamina papyracea, pterygopalatine fossa, and inferior orbital fissure, or vascular extension via embolization, particularly through the ethmoidal veins, leading to intracranial structures like the cavernous sinus.

The underlying causes of immunosuppression predisposing patients to AIFRS are increasingly common due to advances in pharmacological interventions and improved survival rates among immunocompromised patients. In our study, three cases exhibited multiple immunosuppressive comorbidities. In patients with DM, the hyperglycemic environment and ketoacidosis contribute to immune dysfunction by impairing the detection and phagocytosis of fungal pathogens [1,9]. The correction of this impaired immune response is typically more feasible, which could explain the higher survival rates seen in fungal infections in the context of DM [10]. However, other studies have not found a direct correlation between comorbidities and infection prognosis [7,11].

In cases involving hematologic malignancies, neutropenia is the most critical factor. In suspected fungal infections, a reduced absolute neutrophil count is a strong indicator for biopsy, even when there are no suspicious endoscopic findings [4,5]. Payne et al. suggested that in suspected cases of AIFRS with neutrophil counts less than 500/μL, a biopsy of the middle turbinate should be performed even in the absence of endoscopic abnormalities [4]. Additionally, the neutrophil count has prognostic significance, with lower values associated with worse outcomes [5,11].

Imaging studies, particularly CT and MRI, are essential for assessing the extent of infection, planning treatment, and estimating prognosis. In immunosuppressed patients, imaging should be considered even in the absence of suspicious endoscopic findings [5]. However, imaging findings are not pathognomonic, often revealing nonspecific changes such as mucoperiosteal thickening or bone erosion, which typically appear later in the disease course. Extension of the infection into adjacent structures like the orbit or intracranial space is associated with a poorer prognosis [7,11], although the literature is not unanimous in this regard [10]. Wandell et al. reported that cavernous sinus invasion was the only extension associated with significantly worse survival, particularly in patients with DM [10].

The decision to perform orbital exenteration in AIFRS remains undefined. Some authors advocate for this procedure, arguing that the orbit can act as a fungal reservoir where ischemic tissues create a favorable environment for fungal spread. Extensive surgical debridement could prevent intracranial involvement [8]. However, other studies suggest that this high-morbidity procedure does not improve survival and may remove a natural tissue barrier [12]. In our sample, four patients underwent orbital exenteration, none of whom died from fungal infection, raising the question of whether aggressive surgical debridement significantly impacts survival or primarily serves to prevent local complications. While certain clinical signs, such as progressive orbital necrosis, lack of response to antifungal therapy, and severe functional impairment, may suggest consideration of orbital exenteration, our data and the available literature are insufficient to define standardized criteria, underscoring the need for further studies.

In our cohort, *Aspergillus* spp. and *Mucorales* spp. were the predominant pathogens, which is in accordance with the literature [3]. Identifying the specific pathogen is crucial for selecting the appropriate antifungal therapy and for prognostication. *Mucorales* spp. is often associated with a worse prognosis [7,11]. Additionally, rare pathogens, such as *Scedosporium* spp., can be linked to poor outcomes, as seen in a young patient with breast cancer in the present study, who had not yet started chemotherapy and ultimately died.

The identification of the fungal agent through histopathology and microbiology allows for more tailored antifungal therapy, reducing the risk of adverse effects from broad-spectrum antifungals. Correcting neutropenia and controlling hyperglycemia are essential pillars of treatment, as these factors are closely linked to patient survival [1,3,13]. Adjunctive treatments such as granulocyte colony-stimulating factor (G-CSF), granulocyte macrophage colony-stimulating factor (GM-CSF), granulocyte transfusions, and HOT are also relevant in specific cases, particularly those involving neutropenia or hyperglycemia [1,10]. Among the patients in our study, one with a hematologic malignancy and another with diabetes mellitus, each infected with different fungal agents, both received HOT and survived. This therapy may become an increasingly valuable tool in the management of cases with different etiologies and underlying fungal agents, as demonstrated by our study.

Our overall and infection-specific mortality rates were 53.8% and 38.5%, respectively, which are consistent with the ranges reported in the literature [3,13]. The complex clinical picture in these cases, including immunosuppression, oncologic diseases, and vascular pathology, contributes to the high mortality rates. Mortality is likely influenced by multiple factors, including hematologic malignancy or recent bone marrow transplantation/chemotherapy, as well as the extent of infection [10]. Other factors with less direct associations with mortality include the ability to quickly perform surgery, the presence of neutrophilia, and elevated glycated hemoglobin [5,10]. One study reported a disease-specific mortality rate of 14.6%, emphasizing the importance of early diagnosis to intervene while the infection is still localized [4]. However, the underlying clinical condition itself may lead to death in some infected patients. In our sample, two patients died from non-infectious causes: one from progression of the underlying hematologic malignancy and another from a myocardial infarction in the context of unfavourable micro- and macrovascular conditions.

The strengths of this study include the strict histopathological confirmation of AIFRS, which enhances diagnostic accuracy, and the comprehensive characterization of cases, encompassing clinical, microbiological, surgical, and outcome data. The multidisciplinary management approach reflects real-world clinical practice. However, certain limitations must be acknowledged. The small sample size, inherent to the rarity of AIFRS, limits statistical analysis and generalizability. The retrospective single-center design introduces potential selection bias, and the absence of predefined criteria for interventions such as orbital exenteration or HOT reduces reproducibility. Variability in antifungal regimens and the lack of a comparator group prevent definitive assessment of the impact of specific interventions. 

Our findings underscore the importance of early multidisciplinary intervention. In particular, the use of HOT in selected cases with different underlying etiologies or fungal pathogens may improve survival, suggesting a need for further studies to standardize its indications. Additionally, our study highlights the necessity of defining clearer surgical criteria for orbital exenteration, balancing the risks of aggressive debridement with functional preservation. Larger, prospective multicenter studies are needed to define surgical criteria and assess the role of adjunctive therapies in improving survival.

## Conclusions

AIFRS remains a severe and potentially fatal infection, with high mortality rates particularly among immunocompromised patients, such as those with hematologic malignancies, poorly controlled DM, or receiving immunosuppressive therapy. In this study, orbital involvement was frequent, and exenteration was undertaken in select patients. HOT was used as an adjunct in three patients, all of whom survived. Establishing well-defined surgical criteria is essential to optimize outcomes in cases with orbital fungal invasion, underscoring the importance of timely and, when appropriate, aggressive surgical management. Adjunctive therapies, such as HOT, may provide additional benefit and merit further investigation. Larger, prospective studies are required to inform therapeutic decision-making and improve outcomes in this aggressive, rapidly progressive infection.
